# P-1493. *In Vitro* Activity of Clindamycin in Comparison with Linezolid Versus *Streptococcus pyogenes* Clinical Isolates Recovered from Patients in Manitoba, Canada

**DOI:** 10.1093/ofid/ofae631.1663

**Published:** 2025-01-29

**Authors:** Jeremy Li, Philippe Lagace-Wiens, Heather Adam, Peter Pieroni, George Zhanel, James Karlowsky, Andrew Walkty

**Affiliations:** University of Manitoba, Winnipeg, Manitoba, Canada; University of Manitoba, Winnipeg, Manitoba, Canada; University of Manitoba, Winnipeg, Manitoba, Canada; Shared Health, Winnipeg, Manitoba, Canada; University of Manitoba, Winnipeg, Manitoba, Canada; University of Manitoba, Winnipeg, Manitoba, Canada; University of Manitoba, Winnipeg, Manitoba, Canada

## Abstract

**Background:**

Clindamycin is often used in combination with a beta-lactam antimicrobial for the treatment of serious infections caused by *Streptococcus pyogenes* (e.g., necrotizing fasciitis, streptococcal toxic shock syndrome) due to its ability to suppress bacterial toxin synthesis. Of concern, increasing clindamycin resistance among *S. pyogenes* isolates has been reported in many parts of the world. The purpose of this study was to compare the *in vitro* activity of clindamycin with linezolid versus a contemporary Canadian collection of *S. pyogenes* clinical isolates.

**Methods:**

*S. pyogenes* isolates from wounds and sterile body sites (one per patient) were obtained from three microbiology laboratories in Manitoba, Canada between December, 2022 and November, 2023. Susceptibility testing was performed by broth microdilution as described by CLSI, with MICs interpreted using current CLSI breakpoints. A D-test was set up for each isolate to investigate whether inducible clindamycin resistance was present.

**Results:**

Susceptibility testing was performed on 140 *S. pyogenes* clinical isolates. The isolates were obtained from wound swabs (n = 71, 50.7%), sterile fluid/tissue samples (n = 27, 19.3%), and blood cultures (n = 42, 30.0%). All isolates were fully susceptible *in vitro* to penicillin and vancomycin. The proportion of isolates testing susceptible to clindamycin and linezolid was 82.9% and 100%, respectively. All of the clindamycin-resistant isolates demonstrated an inducible resistance phenotype (clindamycin MIC ≤0.12 ug/mL in all cases). Clindamycin susceptibility was similar for isolates from wound swabs (81.7%) and isolates from blood/sterile sites (84.1%).

**Conclusion:**

The proportion of *S. pyogenes* isolates testing susceptible to clindamycin was 82.9%. All clindamycin resistant isolates demonstrated an inducible resistance phenotype. Further data are required to determine whether inducible clindamycin resistance is clinically relevant when clindamycin is used as adjunctive therapy (in combination with a beta-lactam) for serious *S. pyogenes* infections. All isolates tested remained susceptible to linezolid.

**Disclosures:**

**All Authors**: No reported disclosures

